# Presence and Seeding Activity of Pathological Prion Protein (PrP^TSE^) in Skeletal Muscles of White-Tailed Deer Infected with Chronic Wasting Disease

**DOI:** 10.1371/journal.pone.0018345

**Published:** 2011-04-01

**Authors:** Martin L. Daus, Johanna Breyer, Katja Wagenfuehr, Wiebke M. Wemheuer, Achim Thomzig, Walter J. Schulz-Schaeffer, Michael Beekes

**Affiliations:** 1 P24 - Transmissible Spongiform Encephalopathies, Robert Koch-Institut, Berlin, Germany; 2 Prion and Dementia Research Unit, Department of Neuropathology, University Medical Center Göttingen, Göttingen, Germany; Creighton University, United States of America

## Abstract

Chronic wasting disease (CWD) is a contagious, rapidly spreading transmissible spongiform encephalopathy (TSE), or prion disease, occurring in cervids such as white tailed-deer (WTD), mule deer or elk in North America. Despite efficient horizontal transmission of CWD among cervids natural transmission of the disease to other species has not yet been observed. Here, we report for the first time a direct biochemical demonstration of pathological prion protein PrP^TSE^ and of PrP^TSE^-associated seeding activity, the static and dynamic biochemical markers for biological prion infectivity, respectively, in skeletal muscles of CWD-infected cervids, i. e. WTD for which no clinical signs of CWD had been recognized. The presence of PrP^TSE^ was detected by Western- and postfixed frozen tissue blotting, while the seeding activity of PrP^TSE^ was revealed by protein misfolding cyclic amplification (PMCA). Semi-quantitative Western blotting indicated that the concentration of PrP^TSE^ in skeletal muscles of CWD-infected WTD was approximately 2000-10000 -fold lower than in brain tissue. Tissue-blot-analyses revealed that PrP^TSE^ was located in muscle-associated nerve fascicles but not, in detectable amounts, in myocytes. The presence and seeding activity of PrP^TSE^ in skeletal muscle from CWD-infected cervids suggests prevention of such tissue in the human diet as a precautionary measure for food safety, pending on further clarification of whether CWD may be transmissible to humans.

## Introduction

Prions, proteinaceous infectious particles, are the causative agents of transmissible spongiform encephalopathies (TSEs) [1], [2], fatal neurodegenerative disorders of the central nervous system (CNS) such as scrapie in sheep and goats, bovine spongiform encephalopathy (BSE) in cattle, chronic wasting disease (CWD) in cervids or Creutzfeldt-Jacob disease (CJD) and its variant form (vCJD) in humans [3].

Prions are thought to consist essentially – if not entirely – of host-encoded prion protein (PrP) with a pathological folding and aggregation structure, referred to as PrP^Sc^ or PrP^TSE^ [2], [4]. Substantial evidence suggests that the replication of prions is mediated by a process of seeded polymerization [5]. In this process PrP^TSE^ particles (that may or may not contain further components or obtain assistance by helper molecules) exert a proteinaceous seeding activity by putatively acting as nuclei which recruit cellular prion protein (PrP^C^) and incorporate it, in a beta-sheet rich amyloid form, into growing aggregates of misfolded PrP. Fragmentation of aggregates with a critical size eventually mediates the multiplication of particles with proteinaceous seeding activity, resulting in autocatalytic replication of the pathological protein state. PrP^TSE^ has been empirically established as a static biochemical infectivity marker for several different TSE agents under defined analytical conditions, while prion replication by seeded polymerization would conceptually implicate the seeding activity of PrP^TSE^ as the dynamic biochemical correlate to biological prion infectivity [6], [7]. Protein misfolding cyclic amplification (PMCA), which models seeded polymerization of PrP *in vitro* has been established during the past few years as a powerful tool for the ultra-sensitive detection of minute amounts of PrP^TSE^ and PrP^TSE^-associated seeding activity [8], [9].

In individuals with experimentally induced or naturally acquired TSEs prion infectivity and PrP^TSE^ predominantly accumulate in the CNS but were also found, to varying degrees, in peripheral tissues [2], [10]–[12]. Together with CJD and scrapie CWD is one of three naturally occurring forms of prion disease [13]. CWD is contagious and has shown an increasing spread during the past few years in captive as well as free ranging cervids in North America [14]. This has raised concerns for risks of zoonotic CWD transmission to humans via foodstuffs from cervids – not least because the question of whether CWD prions have the ability to infect humans has not yet been definitely resolved. Skeletal muscles from farm- and game animals provide a frequent component of the human diet, and muscle tissue from scrapie-infected mice, hamsters and sheep, or from humans with classical or variant CJD has been previously shown to contain TSE infectivity and/or PrP^TSE^ [10], [11], [15]–[17]. In 2006, Angers et al. [18] reported the detection of prion infectivity in specimens of skeletal muscle from CWD-affected mule deer by bioassay. Although such muscle samples induced disease in reporter animals, PrP^TSE^ or its proteinaceous seeding activity could not be detected directly in skeletal muscles of CWD-infected cervids so far [19]–[21].

Therefore, we applied a high-yield method for the extraction of PrP^TSE^ from muscle samples of CWD-infected WTD and screened the extracts from different skeletal muscles by highly sensitive Western blotting for the presence of PrP^TSE^. In addition to farmed WTD culled in Saskatchewan, Canada, we also analyzed hunter-killed game WTD from Wisconsin, USA. All farmed and free-ranging WTD examined in our study (except for the donor animal of negative control samples) had been officially diagnosed positive for CWD infection by the responsible authorities based on an analysis of brain and/or lymphatic tissue for the presence of PrP^TSE^, although no clinical signs of CWD had been recognized in these animals. In our study, we detected, for the first time, PrP^TSE^ and PrP^TSE^-associated seeding activity in skeletal muscles of farmed and free-ranging cervids infected with CWD. Tissue-blotting revealed that PrP^TSE^ was located in muscle-associated nerve fascicles while PrP^TSE^ was not detectable in mycoytes.

## Results

### Detection and semi-quantitative assessment of PrP^TSE^ in skeletal muscles of CWD-infected white-tailed deer by Western blotting

**Figure 1:**
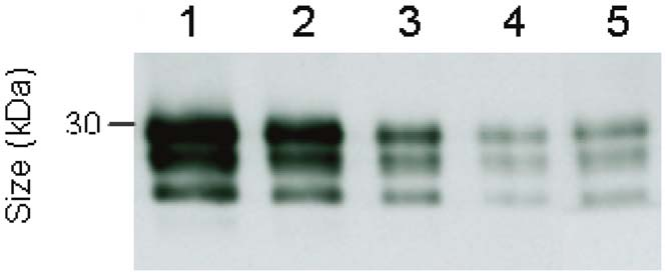
Recovery of PrP^TSE^ after extraction from muscle tissue. Western blot detection of PrP27-30, the Proteinase K-resistant core of PrP^TSE^, after labelling with anti-PrP monoclonal antibody ICSM-18. Lanes 1–4; Proteinase K-digested brain homogenates from WTD in the clinical stage of CWD representing 5×10^−5^ g (lane 1), 1×10^−5^ g (lane 2), 5×10^−6^ g (lane 3) and 1×10^−6^ g (lane 4) of CWD brain tissue. Lane 5; PrP^TSE^ extract from 100 mg of skeletal muscle (*M. glutaeobiceps*) from an uninfected control WTD (blotted also in lane 9 of figure 2; Fa-WTD 10) spiked before extraction with 1×10^−5^ g of CWD brain tissue from a WTD in the clinical stage of disease.

As evidenced by semi-quantitative Western blotting CWD-associated PrP^TSE^ could be recovered, using our extraction procedure, with a yield of approximately 20% from cervid muscle tissue that had been spiked with CWD brain homogenate from WTD in the clinical stage of disease (figure 1).

**Figure 2:**
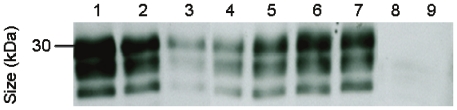
Detection and semi-quantitative assessment of PrP^TSE^ in skeletal muscles of CWD-infected WTD. Western blot detection of PrP27-30, the Proteinase K-resistant core of PrP^TSE^, after labelling with anti-PrP monoclonal antibody ICSM-18. Lanes 1–3, PrP^TSE^ extracts from 100 mg of skeletal muscle from an uninfected control WTD (blotted also in lane 9) spiked before extraction with 1×10^−4^ g (lane 1), 5×10^−5^ g (lane 2), and 1×10^−5^ g of CWD brain tissue from a WTD in the clinical stage of disease. Lanes 4–8, PrP^TSE^ extracts from 100 mg samples of the following muscles from CWD-infected WTD: *M. glutaeobiceps* (farmed animal Fa-WTD 8, lane 4), *M. semimembranosus/tendinosus* (farmed animal Fa-WTD 1, lane 5), *M. semimembranosus/tendinosus* (hunter-kill animal FR-WTD 1, lane 6), *M. semimembranosus/tendinosus* (hunter-kill animal FR-WTD 2, lane 7), *M. semimembranosus/tendinosus* (farmed animal Fa-WTD 2, lane 8). Lane 9, extract from 100 mg sample of *M. glutaeobiceps* from an uninfected control WTD (Fa-WTD 10).

By using this procedure for the extraction of PrP^TSE^ 13 skeletal muscle samples taken from WTD previously found to be infected with CWD were tested for the presence of PrP^TSE^ (table 1). Three out of four donor animals were positive for pathological prion protein PrP^TSE^ in the *M. semimembranosus/tendinosus*, whereas three out of nine animals showed PrP^TSE^ in *M. glutaeobiceps* in the Western blot. This may be indicative of a more pronounced accumulation of PrP^TSE^ in the *M. semimembranosus/tendinosus* as compared to the *M. glutaeobiceps*. However, it has to be noted that the stage of CWD progression was unknown, and possibly differed, for the examined muscle donors. Selected Western blot findings from our study are shown in figure 2. Positive results for farmed cervids are displayed in lanes 4 and 5, while PrP^TSE^ detection in muscle specimens from hunter-kill animals is shown in lanes 6 and 7. Although the donor animal of the muscle sample blotted in lane 8 was previously found to be positive for PrP^TSE^ in brain tissue, too, no PrP^TSE^ could be detected in the examined muscle sample. Negative control muscle from uninfected WTD did not show any PrP^TSE^ staining in independently repeated analyses as exemplified in lane 9.

**Table 1:** Presence and seeding actvity of PrP^TSE^ in skeletal muscles from CWD-infected white-tailed deer.

Donor animal	Clinical symptoms	Official test for CWD infection†	Analysis of M. semimembranosus/M. tendinosus		Analysis of M. glutaeobiceps	
			WB	SA	WB	TB
**FR-WTD 1**	−	**+**	**+**	**n.e.**	**n.a.**	**n.a.**
**FR-WTD 2**	−	**+**	**+**	**n.e.**	**+**	**+**
**Fa-WTD 1**	−	**+**	**+**	**+++**	**−**	**n.e.**
**Fa-WTD 2**	−	**+**	**−**	**+**	**n.a.**	**n.a.**
**Fa-WTD 3**	−	**+**	**n.a.**	**n.a.**	**−**	**n.e.**
**Fa-WTD 4**	−	**+**	**n.a.**	**n.a.**	**−**	**n.e.**
**Fa-WTD 5**	−	**+**	**n.a.**	**n.a.**	**−**	**n.e.**
**Fa-WTD 6**	−	**+**	**n.a.**	**n.a.**	**−**	**n.e.**
**Fa-WTD 7**	−	**+**	**n.a.**	**n.a.**	**−**	**n.e.**
**Fa-WTD 8**	−	**+**	**n.a.**	**n.a.**	**+**	**n.e.**
**Fa-WTD 9**	−	**+**	**n.a.**	**n.a.**	**+**	**n.e.**
**Fa-WTD 10**	−	−	**n.a.**	**n.a.**	−	−

**Footnote to** **Table 1** **.** Explanation of abbreviations: n.a. – no tissue available for testing; n.e. – not examined; SA – PMCA testing for seeding activity of PrP^TSE^; TB – Tissue blot testing for PrP^TSE^; WB – Western blot testing for PrP^TSE^. Explanation of symbols:

† Western blot testing of WTD brain tissue and/or lymph nodes for PrP^TSE^ was performed by the responsible authorities in Canada or the USA in the context of CWD surveillance.

Staining intensities for PrP27-30, the Proteinase K-resistant core of PrP^TSE^, in extracts from test samples representing 100 mg each of muscle tissue from CWD-infected WTD were approximately in the range of signal intensities produced by reference samples containing 10–50 µg of CWD brain tissue from a WTD in the clinical stage of disease (figure 2, lanes 4–7 vs. lanes 2&3). However, the amount of PrP^TSE^ in tested samples varied for different skeletal muscles and animals. A semi-quantitative assessment based on the Western blot findings shown in figure 2 would suggest an about 2000–10000- fold lower concentration of PrP^TSE^ in the tested muscle tissues as compared to CWD brain tissue.

### Proteinaceous seeding activity of muscle-associated PrP^TSE^

The presence of PrP^TSE^ evidenced by Western blotting suggested a contamination of muscle specimens with infectious CWD prions. This prompted us to further examine selected muscle samples for the presence of PrP^TSE^-associated seeding activity, the putative dynamic biochemical correlate of biological prion infectivity (table 1).

**Figure 3:**
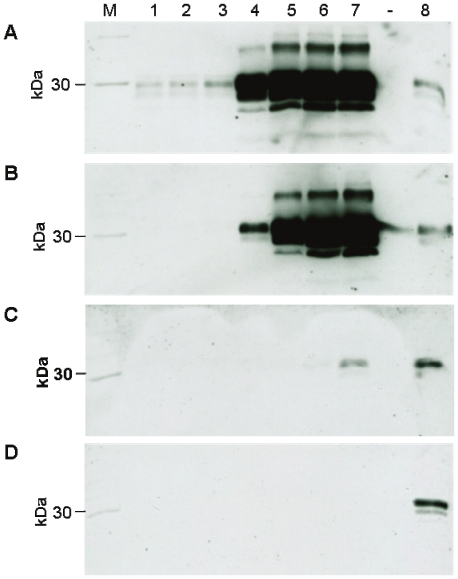
PrP^TSE^-associated seeding activity in muscles from CWD-infected WTD. Western blot detection of Proteinase K-resistant prion protein (PrPres) after PMCA. For PMCA normal hamster brain homogenate was seeded with 10 µl of a 10% (w/v) CWD brain homogenate from an infected WTD (A), or with PrP^TSE^-extract from *M. semimembranosus/tendinosus* of a CWD-infected WTD (Fa-WTD 1, B). In (C) PMCA was seeded with *M. semimembranosus/tendinosus* from Fa-WTD 2 that had been tested positive for PrP^TSE^ in brain tissue while no PrP^TSE^ could be detected by Western blotting in muscle extracts of this animal (the negative result with this muscle is shown in figure 2, lane 8). (D) Unseeded control in which PMCA was performed with normal hamster brain homogenate only. Lanes 1–7 represent Western blot findings after 1, 2, 3, 4, 5, 6 and 7 rounds of PMCA. Lane 8, Proteinase K-digested brain homogenate from a hamster in the clinical stage of scrapie representing 1×10^−7^ g of hamster brain tissue (loaded as an internal blotting control). M: molecular mass marker. Anti-PrP monoclonal antibody 3F4 was used as primary antibody for the detection of PrPres.

By applying a PMCA protocol that used normal hamster brain homogenate as substrate for the *in vitro* amplification of Proteinase K-resistant prion protein (PrPres) muscle extracts from CWD-infected cervids were examined for their seeding activity on PrP^C^. CWD-associated PrP^TSE^ from the brain of infected WTD was found to effectively seed conversion of PrP in normal hamster brain homogenate as shown by PMCA findings such as displayed in figure 3A. Proteinaceous seeding activity was also observed in PrP^TSE^-extracts from muscles of CWD-infected cervids as shown in figure 3B for an extract from *M. semimembranosus/tendinosus* (same sample as blotted in lane 5 of figure 2; Fa-WTD 1).

Figure 3 C displays the result of a PMCA run seeded with a muscle PrP^TSE^-extract for which a direct detection of PrP^TSE^ was not possible by Western blotting (same sample as shown in figure 2, lane 8; Fa-WTD 2). A weak PrPres signal occurred after 7 rounds of PMCA (it has to be noted that this muscle extract was prepared from an animal that had been previously found positive for PrP^TSE^ in the brain). The negative PMCA control (figure 3D) did not produce any detectable PrPres amplification.

### Intramuscular location of PrP^TSE^

The location of PrP^TSE^ in muscle tissue was examined by tissue blotting (and supplementary H&E staining for histomorphological orientation) of sample sections (table 1).

**Figure 4:**
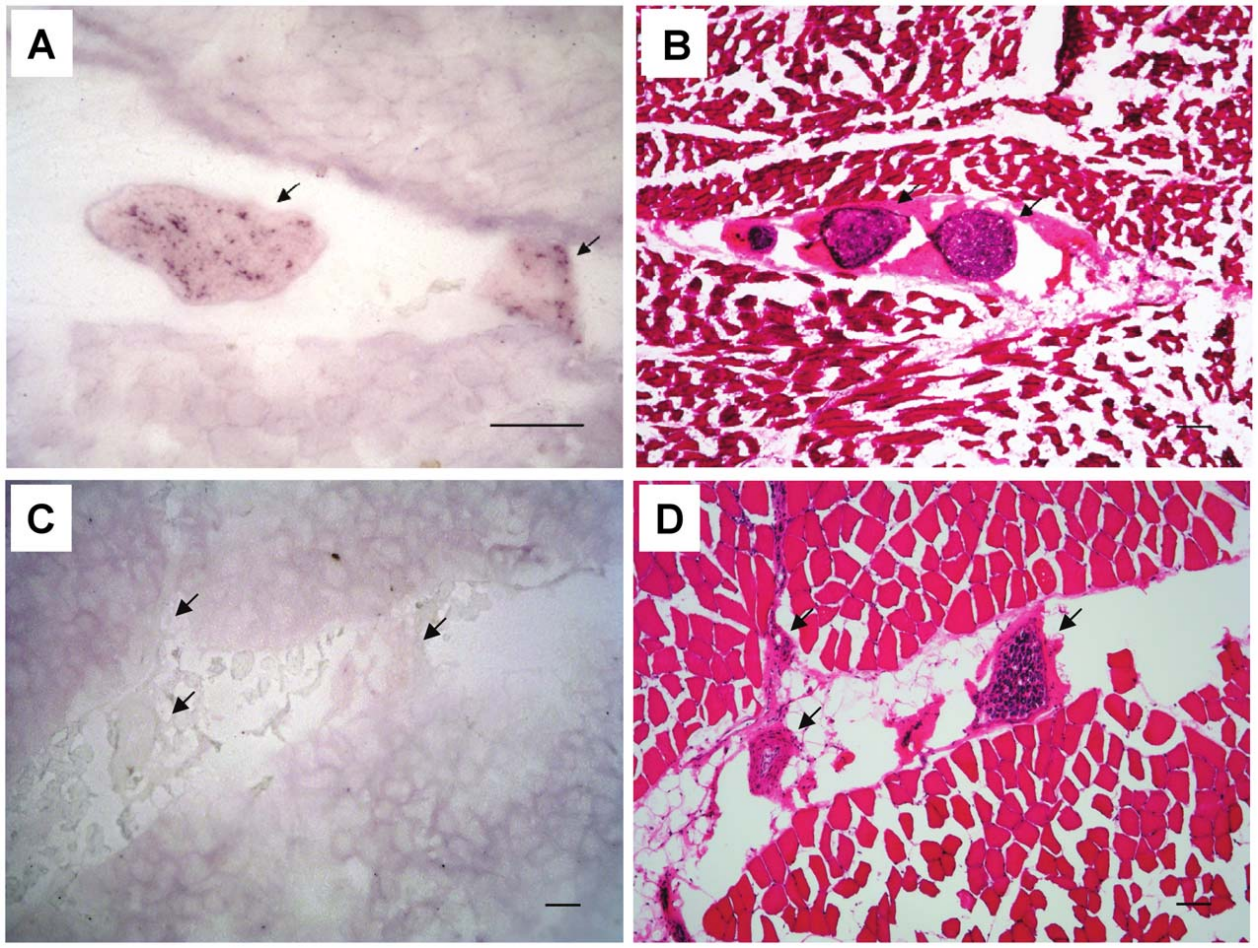
Intramuscular location of PrP^TSE^ in skeletal muscle tissue of CWD-infected WTD. (A) Tissue-blot from *M. glutaeobiceps* of CWD-infected WTD (FR-WTD 2). Distinct granular endoneural PrP^TSE^ accumulation in muscle-associated nerve fascicles visualised by immunolabelling with the monoclonal anti-PrP antibody P4. No PrP^TSE^ accumulation could be detected in muscle fibres. (B) H&E stained slice from the same tissue block as in (A) showing nerves embedded in the muscle tissue. (C) Control tissue-blot of *M. glutaeobiceps* from uninfected WTD (Fa-WTD 10). (D) H&E stained slice from the same tissue block as in (C). Arrows indicate muscle-associated nerve fascicles in A–D. Bars = 2 mm.

Tissue-blotting exclusively revealed deposition of PrP^TSE^ in nerve fascicles while no PrP^TSE^ could be visualized in muscle fibres (i.e. myocytes) from CWD-infected WTD (figure 4A & B; FR-WTD 2). No immunostaining was detectable in tissue blots of muscle tissue from uninfected WTD (figure 4C & D; Fa-WTD 10).

## Discussion

In the wake of the BSE epidemic, variant Creutzfeldt-Jakob disease has emerged as a previously unknown prion disease of humans. This has shown that significant risks for public health may potentially emanate from the presence of animal prions in human foodstuffs. When assessing such risks in the context of CWD, key factors that need to be considered are i) the distribution of CWD prions in infected animals and ii) the potential transmissibility of such prions to humans.

PMCA-based *in vitro* studies have indicated a rather high transmission barrier for CWD to macaques as well as to transgenic mice expressing the human prion protein, as no conversion of cellular prion proteins into PrPres could be induced by seeding PMCA reactions with CWD-infected brain tissue [22]. Consistent with these findings, transgenic mice overexpressing human prion protein with methionine or valine at polymorphic residue 129 were resistant to infection with CWD prions from mule deer [23]. Furthermore, recent *in vivo* studies revealed a robust barrier for the transmission of CWD to macaques which, again, is suggestive of a rather low risk for CWD transmission to humans [24]. However, squirrel monkeys could be infected with CWD [24], and increasing evidence points to the existence of different CWD isolates [25], [26]. Thus, it remains to be further clarified whether CWD may be caused by multiple prion strains with distinct transmission properties.

It has been shown previously that skeletal muscle tissue from CWD infected mule deer may contain prion infectivity [18]. Also, Western blot- and immunohistochemical analyses of heart muscles from CWD-infected Rocky mountain elk and white-tailed deer revealed the presence of pathological prion protein in cardiac myocytes. PrP^TSE^ was found in the left ventricle of the heart at a 10- to 100- fold lower concentration as compared to the brain, but not in skeletal muscle [27].

Here we report the direct detection of pathological prion protein PrP^TSE^ and of PrP^TSE^-associated seeding activity in skeletal muscles from cervids, i. e. WTD for which no clinical signs of prion disease had been recognized, using Western blotting, tissue blotting and PMCA as analytical techniques. Kurt et al. [22] recently reported that normal brain homogenate (NBH) from Syrian hamsters effectively supports the amplification of PrPres from CWD seeds in serial PMCA. Building on similar findings previously made in our laboratory (not shown) we also used hamster NBH as substrate for our PMCA analyses. We found that PrPres generated by PMCA after seeding with CWD- or 263K scrapie agents did not exhibit significant differences in the glycosylation- or electrophoretic migration patterns (not shown). As a safeguard to PMCA specificity safety measures aimed at preventing inadvertent cross-contamination with both prions from cervids or hamsters were implemented. Specifically, we sealed our PMCA reaction tubes with parafilm and paraffin wax, performed all pipetting steps exclusively with plugged single-use pipette tips, and changed gloves each time before handling a new PMCA sample. With these safety measures in place unseeded controls in which PMCA was performed with normal hamster brain homogenate only did not produce any unspecific PrPres staining in our experimental setup.

Our findings were obtained by an alternative methodology to bioassays in animals (i. e. combined biochemical detection of PrP^TSE^ and its proteinaceous seeding activity) but are consistent with the observations reported by Angers et al. [18]. They may also provide an explanation for the negative findings by Jewell et al. [27] in skeletal muscle, since our Western blot results indicate a substantially lower concentration of PrP^TSE^ in such tissue than reported for heart muscle.

Yet, it has to be noted that our assessments of PrP^TSE^ levels in skeletal muscles were based on findings in presumably pre- or subclinically infected animals. Therefore, the concentration of PrP^TSE^ in skeletal muscles of WTD with clinically manifest CWD may possibly exceed our estimate which refers to clinically inconspicuous animals that are more likely to enter the human food chain. Our tissue blot findings in skeletal muscles from CWD-infected WTD would be consistent with an anterograde spread of CWD prions via motor nerve fibres to muscle tissue (figure 4A). Similar neural spreading pathways of muscle infection were previously found in hamsters orally challenged with scrapie [28] and suggested by the detection of PrP^TSE^ in muscle fibres and muscle-associated nerve fascicles of clinically-ill non-human primates challenged with BSE prions [29]. Whether the absence of detectable PrP^TSE^ in myofibers observed in our study is a specific feature of CWD in WTD, or was due to a pre- or subclinical stage of infection in the examined animals, remains to be established. In any case, our observations support previous findings suggesting the precautionary prevention of muscle tissue from CWD-infected WTD in the human diet, and highlight the need to comprehensively elucidate of whether CWD may be transmissible to humans. While the understanding of TSEs in cervids has made substantial progress during the past few years, the assessment and management of risks possibly emanating from prions in skeletal muscles of CWD-infected cervids requires further research.

## Materials and Methods

### Ethics Statement

We did not perform experiments in living animals. However, tissue samples removed *post mortem* from farmed or free-ranging deer and from normal Syrian hamsters were used in our study. All animal work performed in this context was conducted in accordance with relevant national and international guidelines. The farmed or free-ranging deer from which muscle specimens for our analyses were sampled had been culled in the context of livestock farming or deer hunting in Saskatchewan, Canada or Wisconsin, USA. Thus, the removal of tissue samples from these cervids did not require specific approval by animal protection institutions or ethic committees as required for experiments in animals. The sacrifice of normal Syrian hamsters at the Robert Koch-Institut (Berlin, Germany) for the removal of brain tissue to be used in PMCA did not require approval by animal protection instituitions or ethic committees, neither, according to German regulations. However, euthanasia of hamsters for the preparation of PMCA substrate was reported to and registered by the responsible authority (Landesamt für Gesundheit Berlin, Berlin, Germany; http://www.lageso.berlin.de; registration number T0220/07, dated 09 July 2008). Brain tissue from WTD in the clinical stage of CWD used as reference material for Western blotting and PMCA had been sampled in the course of a study previously published by others [30].

### Animal tissues

Specimens from skeletal muscles (*M. semimembranosus/M. tendinosus*; *M. glutaeobiceps*) to be examined by Western blotting or PMCA were taken from farmed or game WTD (*Odocoileus virginianus*) culled in Saskatchewan, Canada (Fa-WTD 1–10) or Wisconsin, USA (FR-WTD 1 & 2), respectively. *M. glutaeobiceps* for tissue blotting was dissected from hunter-harvested animal FR-WTD 2.

At necropsy, WTD torsos with attached extremities were dissected separately from cervid heads. Dedicated sets of instruments were used for the preparation of muscle samples from hind limbs, and there was no contact of instruments or dissected muscle specimens with extra-muscular tissues of the carcass. All muscle tissues tested in our study were prepared under sterile laboratory conditions from inner parts of muscle specimens harvested at necropsy using separate sets of instruments and separate table coverings. Western blot analyses for the presence or absence of detectable PrP^TSE^ in brain tissue and/or lymph nodes from the donor animals of this study were conducted, in the context of CWD surveillance, by the responsible authorities in Canada or the USA. Except for the donor animal of negative control muscle (dissected from unaffected WTD without detectable PrP^TSE^ in lymph nodes or brain [Fa-WTD 10]) all animals we examined for PrP^TSE^ in skeletal muscles had been officially confirmed positive for CWD based on the detection of PrP^TSE^ in lymph nodes or brain (table 1).

For these cervids, however, no clinical signs of CWD had been recognized. Several different symptoms may clinically indicate CWD: Behavioural changes (e. g. reduced interaction with or separation from other animals of the herd, stereotypy, listlessness/apathy, depression, aggressive behaviour, conspicuous excitability), neurological signs (e. g. subtle tremor of the head, ataxia, signs of paralysis, impairment of proprioception, swallowing difficulties, atypical movements of the tongue, teeth grinding), as well as polydipsia, polyuria, excessive salivation, pneumonia, weight loss and wasting. None of these symptoms was reported for the hunted game WTD from Wisconsin (USA). Clinically inconspicuous captive donor animals of our study were culled on a farm in Saskatchewan (Canada) after all WTD showing behaviour or symptoms possibly indicative of CWD (as observed by staff of the Canadian Food Inspection Agency) had been removed from the herd.

The cervid muscle specimens examined in this study were provided by the Saskatoon District Office of the Canadian Food Inspection Agency (Canada) or the Wisconsin Department of Natural Resources (USA), while normal hamster brain tissue was prepared from Syrian hamsters kept at the Robert Koch-Institut in Berlin (Germany). Reference brain tissue from WTD in the clinical stage of CWD was provided by Juergen Richt (Department of Diagnostic Medicine/Pathobiology, College of Veterinary Medicine, Kansas State University, Manhatten, KS, USA).

### Extraction of PrP^TSE^ from muscle samples

For the extraction of PrP^TSE^ from muscle samples we used a previously published protocol [31] with modifications: 100 mg specimens of muscle tissue were cut into small pieces and washed twice in TBS (Tris-buffered saline [10 mM Tris-HCl, 133 mM NaCl, pH 7.4]). Samples were incubated in 900 µl of TBS containing 2 mM CaCl_2_ and 0.25% (w/v) collagenase A (Roche) for 3.5 h at 37°C on a shaking device. For positive controls, muscle tissue from uninfected donors was spiked with PrP^TSE^ by adding defined amounts of brain homogenate from WTD in the clinical stage of CWD. After collagenase A digestion samples were sonicated (Kontes, micro-ultrasonic cell disrupter) and centrifuged at 5.000 rpm (using a TLA-55 rotor) for 3 minutes at 4°C. The supernatant was transferred into a new tube. The pellet was vortexed in 0.5 ml TBS and again centrifuged at 5.000 rpm (TLA-55 rotor) for 3 min at 4°C. The supernatant from the second 5.000 rpm centrifugation was combined with that of the first 5.000 rpm centrifugation and centrifuged for 40 min at 20.000 rpm (TLA-55 rotor) at 4°C. The supernatant was discarded and the pellet resuspended in 1 ml of 1% (w/v) sarcosyl (N-Lauroylsarcosine-sodium-salt [Serva])/TBS by sonication. After centrifugation at 45.000 rpm (TLA-55 rotor) for 2.5 h at 4°C the supernatant was discarded and the pellet resuspended, again by sonication, in 1 ml 0.1% (w/v) sarcosyl, 10% (w/v) NaCl/TBS. 2.5 µg Proteinase K (Boehringer) were added and samples were centrifuged for 2.5 h at 45.000 rpm (TLA-55 rotor) at 4°C. The supernatant was discarded and the pellet was harvested for Western blot detection of PrP^TSE^.

### Western blot detection of PrP^TSE^

For Western blotting final pellets harvested after extraction of PrP^TSE^ from muscle samples were resuspended in 10 µl of distilled water, mixed with an equal volume of 2× electrophoresis sample buffer (4% [w/v] SDS, 10% [v/v] 2-mercaptoethanol in 120 mM Tris-HCl, pH 6.8, containing 20% [w/v] glycerol and 0.05% [w/v] bromphenol blue) and heated to 100°C for 5 min. CWD brain homogenate was digested with 50 µg PK/ml at 37°C for 40 min. SDS-polyacrylamide gel electrophoresis (SDS-PAGE) and Western blot analyses were performed, in principle, as described elsewhere [10] with specific adjustments for the detection of CWD-associated PrP^TSE^: After running SDS-PAGEs using 15% SDS-polyacrylamide gels, proteins were transferred to polyvinylidene difluoride membranes (Immobilon; Millipore) using a semi-dry blot system (Biometra). Membranes were incubated in TBS containing 0.05% Tween (TBST) for 1 h at 20°C. Blots were incubated overnight at 4°C in primary antibody solution (monoclonal anti-PrP antibody ICSM18 [D-Gen], diluted 1 ∶ 10.000 in TBST). After washing three times for 10 min with TBST blots were incubated in secondary antibody solution (alkaline-phosphatase-conjugated goat anti-mouse IgG [DAKO] diluted 1 ∶ 10.000 in TBST) for 90 min at 20°C. After washing three times for 10 min membranes were incubated twice for 5 min in buffer solution (100 mM Tris, 100 mM NaCl, pH 9.5) and subsequently developed with CDP-Star solution (Tropix; Applied Biosystems) for 5 min, according to the manufacturer's instructions. PrP signals were visualised using Amersham Hyperfilm ECL (GE Healthcare).

### Postfixed frozen tissue blotting

Ten-micron frozen sections of muscle tissue (*M. glutaeobiceps*) were cut on a Reichart-Jung cryomicrotome, mounted on nitrocellulose membranes (0.45-mm pore size; Bio-Rad) and fixed for 1 hour in 4% para-formaldehyde in PBS (phosphate buffered saline [4.3 mM Na_2_HPO_4_, 1.4 mM KH_2_PO_4_, 137 mM NaCl, 2.7 mM KCl, pH 7.4]). Protease digestion was performed with 100 µg/ml trypsin (5000 USP-U/mg, salt free; ROTH) in 100 mM Tris-HCl (pH 8.3) at 37°C for 45 minutes. Proteins were denatured by incubation in 4 M guanidine isothiocyanate for 30 minutes. Thereafter, the membranes were further processed according to the paraffin-embedded tissue (PET) blot protocol described elsewhere [32], [33]. Primary monoclonal antibody P4 (R-Biopharm), diluted 1∶5000 in TBST, was used for PrP-detection. Tissue sections from the same block were placed on glass slides and stained with haematoxylin and eosin (H&E) for anatomical orientation.

### Protein Misfolding Cyclic Amplification (PMCA)

PMCA was carried out according to a previously published protocol [34] with modifications: Brains taken from normal Syrian hamsters were homogenized in conversion buffer (PBS [8 mM Na_2_HPO_4_, 1.5 mM KH_2_PO_4_, 137 mM NaCl, 2.7 mM KCl, pH 7.4] containing complete protease inhibitor cocktail [Boehringer-Ingelheim], 4 mM EDTA and 1% Triton-X-100). The normal hamster brain homogenate was adjusted to a concentration of 1 g brain tissue per 10 ml (i. e. to 10% [w/v]), and used as substrate for PMCA. PrP^TSE^-muscle extracts from CWD-infected cervids were prepared according to the protocol described above with modifications: 100 mg of specimens of skeletal muscle were cut into small pieces and washed twice in 1× TBS. Samples were incubated in 900 µl 1× TBS containing 2 mM CaCl_2_ and 0.25% (w/v) collagenase A (Roche) for 3.5 h at 37°C on a shaking device. After incubation at 80°C for 10 min samples were further homogenized by sonication. Samples were centrifuged at 5.000 rpm (using a TLA-55 rotor) for 3 minutes at 4°C. The supernatant was transferred into a new tube. The pellet was vortexed in 0.5 ml TBS and again centrifuged at 5.000 rpm (TLA-55 rotor) for 3 min at 4°C. The supernatant from the second 5.000 rpm centrifugation was combined with that of the first 5.000 rpm centrifugation. The combined supernatants were centrifuged for 2.5 h at 45.000 rpm and 4°C using a TLA-55 rotor. Resulting supernatants were discarded and pellets were resuspended in 100 µl conversion buffer. For the seeding of PMCA with PrP^TSE^ extracts from muscle tissue 20 µl aliquots of resuspended pellets were mixed with 130 µl of 10% (w/v) normal hamster brain homogenate. 140 µl of 10% (w/v) normal hamster brain homogenate were used as substrate when PMCA was seeded, as a positive control, with 10 µl of a 10% (w/v) CWD brain homogenate (that had been similarly prepared as described above for normal hamster brain homogenate) from a WTD in the clinical stage of disease. As a negative control unseeded PMCA was performed only with normal hamster brain homogenate. In order to prevent cross-contamination of samples during PMCA all pipetting steps were performed with plugged single-use pipette tips, and gloves were changed each time before handling a new sample. PMCA was performed in 0.5 ml Eppendorf safe lock tubes that had been subjected to steam sterilization at 121°C and 3 bar for 20 min prior to use and were sealed with parafilm and paraffin wax before PMCA processing. PMCA was carried out using an automatic sonication apparatus (Sonicator S-4000 from Misonix, New York, USA). After each round of PMCA 75 µl aliquots of PMCA batches were transferred into 75 µl of fresh normal hamster brain homogenate. One round of PMCA consisted of 24 alternating cycles of sonication (for 40 sec every 60 min) and incubation of samples at 37°C between sonications. PMCA-batches obtained after 1, 2, 3, 4, 5, 6 and 7 rounds of PMCA were exposed to Proteinase K at a concentration of 150 µg PK/ml and incubated for 1 h at 55°C. After centrifugation for 1 min at 13.000 g and room temperature, 30 µl aliquots of PK-digested PMCA-samples were mixed with 30 µl of 2× electrophoresis sample buffer, each, and heated for 10 min at 100°C. Subsequently, 10 µl aliquots of these samples were subjected to SDS-PAGE and Western blotting. PK-resistant prion protein (PrPres) in PMCA products was labelled with the monoclonal anti-PrP antibody 3F4 [10] which does not recognize CWD-associated PrP^TSE^.
